# Medication-Related Osteonecrosis of the Jaw: New Insights into Molecular Mechanisms and Cellular Therapeutic Approaches

**DOI:** 10.1155/2016/8768162

**Published:** 2016-09-18

**Authors:** Thomas Lombard, Virginie Neirinckx, Bernard Rogister, Yves Gilon, Sabine Wislet

**Affiliations:** ^1^Laboratory of Nervous System Diseases and Therapy, GIGA-Neuroscience, University of Liège, Liège, Belgium; ^2^Department of Maxillofacial Surgery, CHU, University of Liège, Liège, Belgium; ^3^Department of Neurology, CHU, University of Liège, Liège, Belgium

## Abstract

In recent years, medication-related osteonecrosis of the jaw (MRONJ) became an arising disease due to the important antiresorptive drug prescriptions to treat oncologic and osteoporotic patients, as well as the use of new antiangiogenic drugs such as VEGF antagonist. So far, MRONJ physiopathogenesis still remains unclear. Aiming to better understand MRONJ physiopathology, the first objective of this review would be to highlight major molecular mechanisms that are known to be involved in bone formation and remodeling. Recent development in MRONJ pharmacological treatments showed good results; however, those treatments are not curative and could have major side effects. In parallel to pharmacological treatments, MSC grafts appeared to be beneficial in the treatment of MRONJ, in multiple aspects: (1) recruitment and stimulation of local or regional endogenous cells to differentiate into osteoblasts and thus bone formation, (2) beneficial impact on bone remodeling, and (3) immune-modulatory properties that decrease inflammation. In this context, the second objective of this manuscript would be to summarize the molecular regulatory events controlling osteogenic differentiation, bone remodeling, and osteoimmunology and potential beneficial effects of MSC related to those aspects, in order to apprehend MRONJ and to develop new therapeutic approaches.

## 1. Introduction

In 2014, the American Association of Oral and Maxillofacial Surgeons (AAOMS) has updated the definition of bisphosphonate-related osteonecrosis of the jaw (BRONJ) [1], given the increasing number of osteonecrosis of the jaw cases reported in patients treated with bisphosphonates (BPs). However, since this clinical condition is also encountered in patients treated with denosumab or other antiangiogenic drugs [2–4], the term “medication-related osteonecrosis of the jaw” (MRONJ) should be favored. MRONJ is defined by three features: (1) current or previous treatment with antibone resorptive or antiangiogenic agents, (2) exposed bone or bone that can be probed through an intraoral or extraoral fistula in the maxillofacial region that stays for longer than 8 weeks, and (3) no previous history of radiation therapy or obvious metastatic disease towards the jaws [1].

In osteoporotic patients, the incidence of MRONJ is 1.04 to 69 per 100,000 patient-years if treated by oral BPs, 0 to 90 per 100,000 patient-years if treated by i.v. BPs, and 0 to 30,2 per 100,000 patient-years if treated by denosumab [5–7]. In oncologic patients, the incidence of MRONJ is 0 to 12,222 per 100,000 patient-years if treated by i.v. BPs and 0 to 2,316 per 100,000 patient-years if treated by denosumab [5–7]. Risk factors for MRONJ are multiples; the major ones are i.v. BPs (depending on dose and duration), Zoledronate, dental extraction, dental or periodontal disease, glucocorticoid, chemotherapy, smoking, and obesity [8, 9].

MRONJ is two times more frequent in the mandible than in the maxilla [10]. The most accepted clinical staging system for MRONJ has been developed by Ruggiero and colleagues and has been adopted by the AAOMS [1]. This clinical scale describes five stages: at risk, 0, 1, 2, and 3. Stage “at risk” includes patients undergoing treatment with oral or intravenous nitrogen-containing BPs, with no evidence of necrotic bone. Stage 0 includes patients presenting nonspecific clinical findings, radiographic changes, and symptoms with no clinical evidence of bone necrosis. Stage 1 includes asymptomatic patients presenting an exposed and necrotic bone or fistulae. Stage 2 includes symptomatic patients (pain, erythema, and signs of infection) presenting an exposed and necrotic bone or fistulae. Stage 3 includes stage 2 patients with one of the following: (1) bone lesions extending beyond the region of the alveolar bone resulting in pathologic fracture, extraoral fistula, or oroantral/oronasal communication or (2) osteolysis extending to the inferior border of the mandible or sinus floor.

In this review, after considering drugs that have been shown to be responsible of MRONJ, we will briefly comment on current physiopathological hypotheses that could explain this particular clinical situation. We will then review several putative treatments, with a deeper focus on cellular therapy protocols, including (1) drug-based manipulation of bone marrow stem cells and (2) mesenchymal stem cell (MSC) grafts, which are both experimental therapeutic approaches currently used to treat this incapacitating clinical situation. Aiming to better understand MRONJ physiopathology, we will also summarize molecular mechanisms that are known to be involved in bone formation and remodeling, as well as MSC involvement in these processes. Finally, we will discuss the link between bone homeostasis and the immune system, referred to as “osteoimmunology.” Indeed, the MSC effect could also include a modulation of this osteoimmunological homeostasis, explaining their therapeutic effects.

## 2. Drugs-Related Osteonecrosis of the Jaw

Antiresorptive and antiangiogenic drugs were previously shown as implied in the development of MRONJ [2–4] (Table 1). Antiresorptive drugs (bisphosphonate and denosumab) are monoclonal antibodies directed against Receptor Activator of Nuclear Factor Kappa-B Ligand (RANKL). Antiangiogenic drugs (Sunitinib® and Bevacizumab®) are humanized monoclonal antibodies directed against several activated receptors tyrosine kinase (i.e., VEGFR (vascular endothelial growth factor receptor)).

- *Bisphosphonates (BPs)* are used to treat a wide variety of diseases characterized by excessive osteoclast-mediated bone resorption, such as tumor-associated osteolysis, Paget's disease, hypercalcemia of malignancy, and osteoporosis [7]. BPs are stable and nonhydrolysable analogs of pyrophosphates that are composed of a carbon atom linked to two phosphonate groups (P–C–P) to mimic the pyrophosphate molecular structure (P–O–P). Simple BPs (SBPs) (etidronate and clodronate) should be distinguished from nitrogen-BPs (N-BPs) (pamidronate, alendronate risedronate, ibandronate, and Zoledronate) because of the presence of nitrogen on the side chains of the latter. This structural difference has an impact on the mechanism of action. Indeed, in addition to their analog effect, only SBPs are metabolized into intracellular and nonhydrolysable cytotoxic analogs of ATP, which accumulate in the osteoclasts and trigger their apoptosis [17]. In contrast, N-BPs inhibit osteoclast function only by acting as potent inhibitors of the enzyme farnesyl-diphosphate (FPP) synthase in the cholesterol (or mevalonate) biosynthetic pathway. This inhibition is responsible of a decrease of GTPase activity in cytoskeletal rearrangement and vesicular trafficking in osteoclasts [18]. N-BPs might also have an effect on the immune system, especially on macrophages and monocytes, but this effect remains controversial [19]. It has also to be noted that N-BPs are 100 to 10,000 times more potent than SBPs [20]. More recently, it has been demonstrated that BPs (alendronate and Zoledronate®) induce osteogenic gene expression, such as bone morphogenic protein-2 (BMP-2), osteocalcin, and alkaline phosphatase in endothelial and mesenchymal stem cell [21].
- *Denosumab* is a recent antiresorptive drug that showed better results than alendronate in improving bone mineral density in different skeletal sites [22]. This human monoclonal antibody targets the Receptor Activator of Nuclear Factor Kappa-B Ligand (RANKL) [23]. In humans, bone remodeling depends on a balance between osteoprotegerin (OPG) and RANKL that are both produced by osteoblasts (see molecular signaling controlling bone remodeling section below). RANKL binds to its receptor (RANK) expressed by preosteoclasts and osteoclasts and induces, respectively, their full differentiation and activation. OPG and denosumab have the same mechanism of action: they bind to RANKL, then blocking its interaction with RANK, inhibiting the osteoclast maturation, function, and survival, and reducing bone resorption [23].
- An increasing number of MRONJ cases are now reported in patients treated with antiangiogenic drugs [20]. These drugs are VEGF antagonists and might be divided into two categories: (1) monoclonal antibodies that bind VEGF and, thereby, neutralize its biological activity (Bevacizumab) and (2) small molecule tyrosine kinase inhibitors (TKIs) that block the VEGF receptor and its downstream signaling pathways (Sunitinib, Sorafenib®, Cabozantinib®). VEGF antagonists are used to treat metastatic cancer such as renal, colorectal, lung, and breast carcinomas [20]. Inhibitors of mammalian target of rapamycin (mTOR) (Everolimus® and Temsirolimus®) are recent therapeutic agents used in the treatment of metastatic renal carcinoma. These drugs have also been reported as MRONJ inducers in two case reports [24, 25].

## 3. Pathogenesis of MRONJ

**Figure 1:**
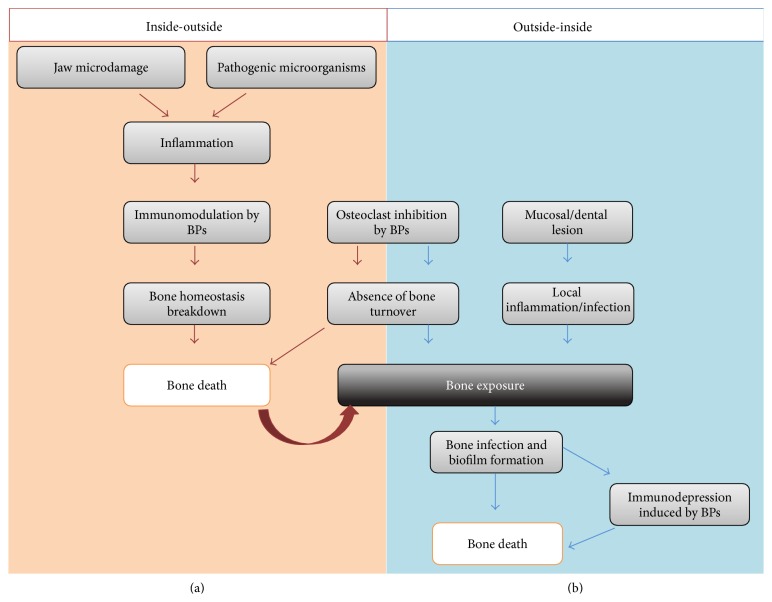
Two MRONJ pathogenesis theories: inside-outside and outside-inside theory.

The pathogenesis of MRONJ has been studied for multiple years but still remains unresolved. However, two theories are emerging (Figure 1). The first one, called “inside-outside,” is based on an inhibition of the osteoclastic activity and a bone turnover decrease, both conditions induced by previously mentioned drugs. This decrease in bone remodeling despite the jaw microdamage induced by chewing and local inflammation results in a constant exposition of bone to high concentrations of various pathogenic microorganisms (Figure 1(a)). These conditions would lead to bone tissue death and then to bone exposure [26]. In various ONJ (Osteonecrosis of the Jaws) animal models, it has been reported several times that most of the specimens present a histological osteonecrosis, but only a minority of them show an exposed bone (not covered by epithelium) [27, 28]. Another histological study of ONJ lesions in humans also concluded that bone necrosis precedes the clinical onset and is then responsible of an inflammation-associated process [29]. All of these findings suggest thus that bone exposure could not be a prerequisite for bone necrosis. Moreover, the fact that two different antiresorptive drugs (BPs and denosumab) with different mechanisms of action are implied into MRONJ underlines the central role played by bone resorption inhibition in mechanically stressed jaws. This possible central role of bone resorption inhibition in the physiopathology of MRONJ is also strengthened by encouraging results reported for Teriparatide® drug, a recombinant human parathyroid hormone that stimulates osteoclast activity [30]. All these observations corroborate the bone resorption inhibition theory as the etiology of MRONJ. However, there is so far no reported case of ONJ in patients with a reduced bone turnover condition, such as hypoparathyroidism.

The second theory, named “outside-inside,” is based on a local immune-depression, probably caused by BPs or denosumab associated with mucosal/dental lesions that would lead to a local infection and/or inflammation spreading to the bone and, there, inducing the osteonecrosis (Figure 1(b)). It has been established that dental diseases are an important risk factor in MRONJ: the efficient prevention of MRONJ in patients with cancer is observed by improvements in their dental hygiene [31]. It is also important to highlight that most of the tooth extractions were done because of an existing periodontal or periapical disease [32]. Recent studies also demonstrated that periodontal or periapical diseases associated with i.v. BPs could cause MRONJ in animal models [27, 28]. Finally, it has been reported that exposed bone areas in MRONJ are recovered by a complex biofilm with multiple microorganisms which could explain therapy failures [33].

The reason why medication-related osteonecrosis specifically affects the jaw is still unknown, but some clues can be pointed out. The jaw is one of the least-protected bones from infection in the human skeleton. Indeed, the mandibular and maxilla bones are just separated from the pathogens of the oral mucosal lesion by a thin mucoperiosteal cover, whereas deep soft tissues and skin protect other bones. Moreover, jaw is subjected to repeated microtraumas due to the presence of teeth and the force of mastication. Indeed, the alveolar bone turnover is 10-fold greater than in the long bones which could justify the fact that alveolar bone could incorporate much more BPs than other skeleton sites [34]. Finally, the jaw has a specific embryologic development. It arises from neural-crest cells which form at the border of the neural tube during neurulation, and not from the mesoderm like other bone cells of the body [35]. Furthermore, in a recent study [36], it has been demonstrated that jaw bone defects could be healed through neural-crest cell recruitment.

For an unknown reason, it appears that tissue homeostasis in the mandibular and maxilla bones is disrupted in MRONJ patients, by the combination of (1) drugs acting more or less on bone turnover, (2) the proximity of a highly septic environment, and (3) the mechanical stress induced by chewing several times a day. This misbalance in tissue homeostasis leads to necrosis, which itself increases and/or maintains this misbalance, triggering a vicious circle. Treatments should therefore address this misbalance by acting on regenerative processes, in attempt to reequilibrate this compromised situation.

A clear understanding of MRONJ pathogenesis is mandatory before considering any therapeutic perspective. We suggest three potential etiologies: (1) the lack of bone formation caused by the absence of osteogenic differentiation from MSC, (2) the imbalance in bone remodeling caused by BPs or denosumab, and (3) the homeostasis disruption between the immune system and bone which refers to the new concept of osteoimmunology. In the next few paragraphs, we will therefore focus on the molecular patterns that underlie these potential etiologies.

### 3.1. Molecular Signaling Pathways Controlling Osteogenic Differentiation

**Figure 2:**
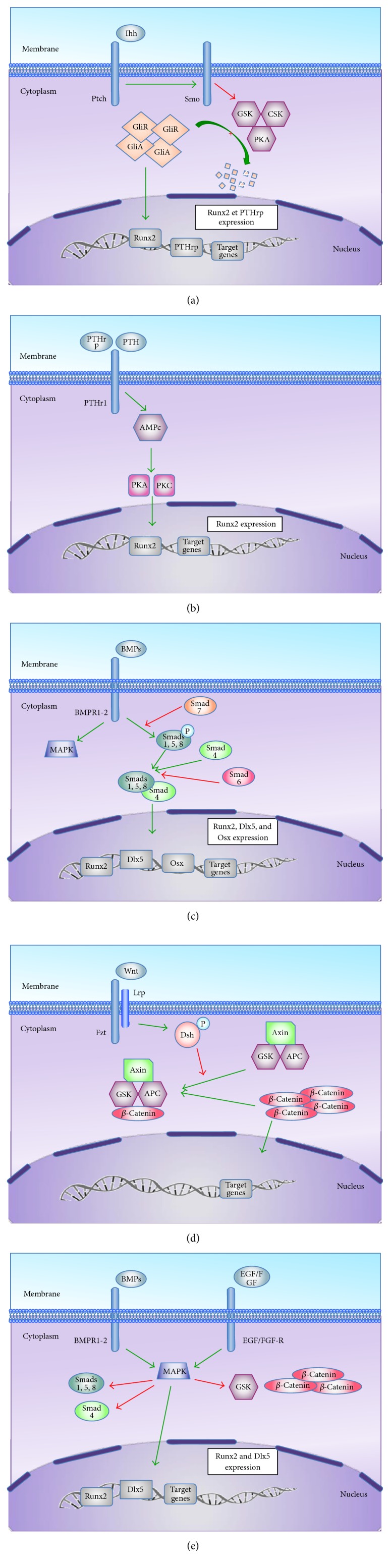
Molecular regulation of MSCs during bone formation. (a)* Ihh pathway*: Indian hedgehog (Ihh) stimulates, directly or indirectly (through the parathyroid hormone-related peptide (PTHrp) synthesis), chondrocytes proliferation and their differentiation into hypertrophic or larger cells. Ihh binds to its receptor Patched (Ptch), which inhibits Smoothened (Smo). The resulting activation of Smo leads to an increase of intracellular concentration of Gli proteins (Gli activator (GliA) and Gli repressor (GliR)) subsequent of the inhibition of their degradation regulated by glycogen synthase kinase (GSK3*β*), protein kinase A (PKA), and casein kinase (CSK). After translocation into the nucleus, Gli activator could bind to its promoter and stimulate various genes' expression, especially Runx2 [11, 12]. (b)* PTH and PTHrp pathway*: parathyroid hormone (PTH) and parathyroid hormone-related (PTHrp) bind to PTH-receptor1 (PTHr1), which is a G protein-coupled receptor that activates adenylate cyclase. This leads to cAMP production, PKA and PKC stimulation, and Runx2 expression. The exact mechanism leading to Runx2 is still unknown. (c)* BMP pathway*: bone morphogenetic proteins (BMPs) binds to a tetrameric receptor encompassing type I (BMPR1) and type II (BMPR2) receptors that are serine-threonine kinases. The receptor activation induces signal transduction through Smads or mitogen-activated protein kinase (MAPK). Smads are cytoplasmic molecules that are classified into 3 subsets: (1) receptor-regulated Smads (Smads 1, 2, 3, 5, and 8); (2) common-partner Smads (Smad 4); (3) inhibitory Smads (Smads 6, 7). Smads 1, 5, and 8 are activated by phosphorylation induced by BMPs interacting with their receptors. Receptor-regulated phosphorylated Smads are then able to form a dimeric complex with Smad 4 allowing its nuclear translocation. When phosphorylated, Smads 6 and 7 both inhibit Smads 1, 5, and 8 phosphorylation and Smad 4 linking [13]. In the nucleus, the dimeric Smad complex will induce the target genes expression such as Runx2, distal-less homeobox 5 (Dlx5), and osterix (Osx) which are osteoblastic genes [14, 15]. (d)* Wnt-β catenin canonical pathway*: Wnt molecules are involved in multiple cell functions, including osteogenesis. Wnt-1, Wnt-3a, Wnt-4, Wnt-5, Wnt-10b, and Wnt-13, are essential in bone formation [16]. Wnt binds to its receptor Frizzled (Fzd) and coreceptor, low-density lipoprotein receptor-related protein (Lrp). In absence of binding, dishevelled (Dsh) remains inactivated in the cytoplasm and *β* catenin can form a complex with GSK3*β*, adenomatous polyposis coli (APC), and axin that leads to their degradation by ubiquitination. When Wnt binds to its receptor, phosphorylated Dsh induces axin and GSK3*β* inhibition and thus leads to *β*-catenin accumulation. *β*-Catenin is then able to translocate into the nucleus where it drives the target genes expression. (e)* MAPK pathway*: mitogen-activated protein kinases (MAPKs) are able to phosphorylate and inhibit GSK3*β* and Smads 1, 5, and 8 activities. They are also able to induce Runx2 and Dlx5 expression. MAPK can be triggered by epithelial growth factor (EGF), fibroblast growth factor (FGF), and BMPs.

During development, bone formation begins with MSCs aggregation and, then, cells differentiate sequentially into chondrocytes and osteoblasts during endochondral ossification [14]. After condensation, MSCs could also directly differentiate into osteoblasts in a process called “membranous bone ossification.” Sox9 and Run-related transcription factor 2 (Runx2) are two essential transcription factors expressed in MSCs during osteoblast differentiation. Sox9 induces cell condensation which is precluding their conversion and differentiation into chondrocytes [37]. Runx2 stimulates chondrocyte proliferation and growth into larger cells and then into osteoblasts [38]. Besides this intracellular signalization, there are five extracellular pathways that are identified in osteoblastogenesis as summarized in Figure 2: (1) Ihh, (2) PTH and PTHrp, (3) BMP, (4) Wnt-*β* catenin canonical, and (5) MAPK pathways.

**Figure 3:**
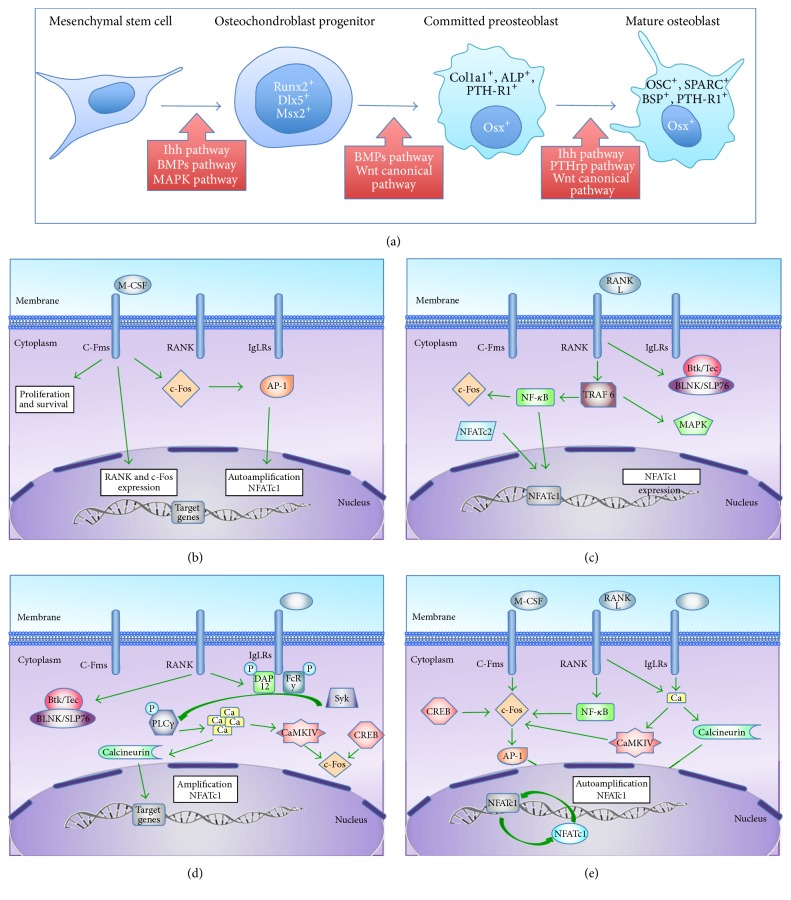
Bone remodeling: (a) in precursor cell stage, the macrophage-colony stimulating factor (M-CSF) binds to its receptor (c-Fms). It promotes survival and proliferation of osteoclast precursors, as well as RANK and c-Fos expression. (b)* c-Fms pathway*: M-CSF binds to c-Fms and promotes cell proliferation and survival. It also promotes RANK and c-Fos expression as well as NFATc1 autoamplification through AP1. (c)* RANK pathway*: the binding of RANKL to RANK promotes the recruitment of tumor necrosis factor receptor-associated factor 6 (TRAF6), which can activate the nuclear factor-*κ*B (NF-*κ*B) and mitogen-activated protein kinases (MAPKs), such as p38 and Jun N terminal kinase (JNK). TRAF6-activated NF-*κ*B induces the expression of NFATc1, an important transcription factor for osteoclastogenesis. This NFATc1 expression is also stimulated by the nuclear factor of activated T cells cytoplasmic 2 (NFATc2). Finally, RANK also activates the tyrosine kinases Btk and Tec that are involved in the phosphorylation of phospholipase C*γ* (PLC *γ*). NF-*κ*B can also stimulate the c-Fos induction. (d)* IgLRs pathway and calcium signaling*: RANK activation leads to phosphorylation of DAP12 and FcR*γ*. These molecules are both associated with IgLRs and stimulate Syk. Activated Btk/Tec/BLNK/SLP76 complex and Syk will phosphorylate PLC-*γ* which will mediate the calcium release from intracellular stores. Calcium will activate calcineurin phosphatase which is involved in NFATC1 autoamplification. It also stimulates C-Fos through CaMKIV activation. (e)* NFATc1 autoamplification*: by these three pathways, AP-1, calcineurin and NFATc1 participate in NFATc1 autoamplification. Indeed, AP-1 and the continuous calcium signaling are essential for NFATc1 amplification. The NFATc1 promoter is epigenetically activated through histone acetylation and contains NFAT binding sites. Thus, NFATc1 specifically autoregulates its own promoter and is responsible for its robust induction. NFATc1 is negatively regulated by other transcription factors, such as IRF8, MafB, and Bcl6 that are, in turn, inhibited by Blimp1, a transcriptional target of NFATc1.

During fetal bone formation, MSCs are recruited. Runx2 expression is then activated, which induces MSCs differentiation into osteochondroblast progenitors [38] (Figure 3(a)). In this early stage, mostly Ihh pathway activates Runx2. At later stage, BMPs and MAPK pathways stimulate Runx2 but also Dlx5 expression. Dlx5 is an osteogenic homeobox protein involved in osteoblasts maturation [39]. Depending on Dlx5 levels, Msx2, another osteogenic homeobox protein, induces immature cell proliferation.

Osteochondroblast progenitors (Runx2^+^, Dlx5^+^, and Msx2^+^ cells) mature into committed preosteoblast cells, which express osterix (Osx), collagen 1*α*1 (Col1*α*1), alkaline phosphatase (ALP), and PTH-R1. Osterix is a transcriptional factor acting as an essential regulator of late osteogenesis through the inhibition of chondrogenesis [40]. This process of maturation for osteochondroblast is induced by BMP and Wnt canonical pathways. Preosteoblast cells (Osx^+^, Col1*α*1^+^, ALP^+^, and PTH-R1^+^ cells) are then maturing in osteoblasts. On the one hand, this maturation is due to Wnt canonical pathway. On the other hand, osteochondroblast progenitors (Runx2^+^, Dlx5^+^, and Msx2^+^ cells) are able to secrete Ihh, which induces PTHrp production. Both molecules participate in preosteoblastic cell maturation. Mature osteoblast expresses specific bone proteins such as osteocalcin (OSC), bone sialoprotein (BSP), PTH-R1, and osteonectin [14].

Based on all these molecular pathways, various therapeutic approaches were investigated:

(i) *Teriparatide and BMPs*, as described below, were used to stimulate the bone formation and to treat MRONJ [41, 42].
(ii) *Dickkopfs 1 (DKK1)* is a natural Wnt-antagonist that binds Lrp5/6. DKK1^+/−^ mice show an increase in all bone formation parameters [43]. By inhibiting DKK1, another study has observed an increase of bone density in a multiple myeloma mouse model [44].
(iii) Another study reported that MSCs graft coupled with lithium chloride treatment, a* GSK3β inhibitor*, stimulates their differentiation into osteoblast* in vivo* and* in vitro* [45].
(iv) *Sclerostin* is another Wnt-antagonist that binds Lrp5/6, but in a different region from DKK1. Li et al. (2009) reported that antisclerostin antibody treatment increases bone formation and bone mass in a rat model of osteoporosis [46].

### 3.2. Molecular Signaling Controlling Bone Remodeling

Even in adulthood, bone remains a highly dynamic organ in constant remodeling. Two principal actors take part in bone remodeling processes: (1) MSC-derived osteoblasts, which promote bone formation and osteoclast, and (2) CD34+ hematopoietic progenitor-derived osteoblasts, which promote bone resorption. The bone remodeling balance between osteoformation and osteoresorption is regulated by several cytokines. The most characterized mechanism is the balance between osteoprotegerin (OPG) and the Receptor Activator of Nuclear Factor Kappa-B Ligand (RANKL), which are both expressed and secreted by osteoblasts. As an autocrine factor, RANKL binds to its transmembrane receptor (RANK) present on preosteoclasts and osteoclasts and induces their differentiation and activation, respectively. The balance is due to OPG that binds to RANKL, blocking its interaction with RANK and, thus, inhibiting the osteoclast maturation, function, and survival. The balance consequently tips towards reducing bone resorption in presence of OPG [23]. Globally, RANK activation leads to the expression and activation of nuclear factor of activated T cells cytoplasmic 1 (NFATc1). The primordial and sufficient role of this transcription factor in osteoclastogenesis has been demonstrated* in vitro* and* in vivo* [47].

The signaling pathway during osteoclastogenesis is based on three receptors: c-Fms, RANK, and Immunoglobin-Like Receptors (IgLRs) (OSCAR, PIR-A, SIRP*β*1, and TREM 2), as described in Figures 3(b)–3(e).

### 3.3. Molecular Signaling Controlling Osteoimmunology

During the last decade, the involvement of immunological cells and cytokines in bone remodeling took a greater place. For example, OPG is expressed by B cells and dendritic cells [48]. RANKL is expressed by B cells, T cells, and *γδ*-T cells, while RANK is expressed by macrophages and monocytes [49]. Besides these examples of bone cytokines playing a role in immune system, it was also demonstrated that several immune cytokines could modulate the bone biology: (1) important inflammatory cytokines such as IL-1, IL-6, and TNF-*α* stimulate RANKL expression and accelerate bone destruction and (2) a variety of cytokines such as IFN-*γ*, granulocyte/macrophage-colony stimulating factor (GM-CSF), IL-4, and IL-10 were shown to stimulate bone formation [50].

**Figure 4:**
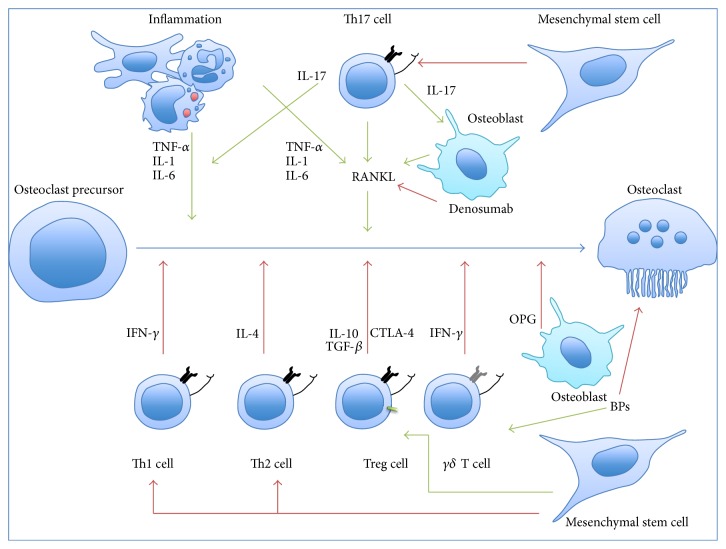
Osteoimmunology: differentiation of osteoclast precursor into mature osteoclast. Synthesis of osteoblast, immune, and mesenchymal cell action in osteoclastogenesis: (1)* T helper cells type 1 (Th1)* that are playing a role in cellular immunity, are induced by IL-12, and secrete IL-2 and INF-*γ* (which has antiosteoclastogenic properties). (2)* T helper cells type 2 (Th2)* are involved in humoral immunity. They are induced by IL-4 and secrete IL-4 and IL-13. IL-4 has also antiosteoclastogenic properties. (3)* The T helper cell type 17 (Th17)* differentiates from naïve T CD4+ cells, has a proinflammatory role, and is implicated into autoimmune disease. Th17 is induced by TGF-*β*, IL-6, IL-21, and especially IL-23. Th17 cells secrete IL-17, IL-21, and IL-22. IL-17 is a major inflammatory cytokine and IL-21 stimulates Th17 differentiation and inhibits Th1 and Treg cells actions. In osteoclastogenesis, IL-17 can produce and induce RANKL expression by osteoblast, a situation that favors osteoresorption. This is not the only stimulatory activity of Th17 on osteoresorption as these cells express also higher levels of RANKL compared to Th1 and Th2. Finally, they also have higher levels of IL-1, IL-6, and TNF-*α*.

Unraveling bone homeostasis regulation allows for highlighting connections between bone remodeling and immune cells. The role of T cells in osteoclastogenesis was more specifically analyzed and summarized in Figure 4. Looking at that figure, we could conclude that Th17 cells are the link between bone destruction and the immune system. On the other hand, if Th17 are the immune cells responsible for the stimulation of osteoclastogenesis, regulatory T cells or Treg should be regarded as immune cells that stimulate bone formation by downregulation of osteoresorption. Treg are CD4^+^CD25^+^FOXP3^+^ cells that are specialized in tolerance, immunity inhibition, autoimmune pathology prevention, and regulation of inflammation [51]. Treg also express specific surface molecules including GITR and CTLA-4 [52]. Noteworthily, FoxP3 has been described as specific and mandatory for the development and activity of Treg cells [53]. These cells are also known to secrete IL-10 and TGF-*β*, which both trigger reduction of inflammation and bone destruction and have an inhibitory effect on osteoclastogenesis [54]. However, controversial studies reported that IL-10, IL-4, and TGF-*β* have the higher antiosteoclastic effects. Globally, TGF-*β*, IL-4, and IL-10 are potent antiosteoclastic cytokines, but further studies are mandatory to understand their mechanisms of action [55]. Treg cells are also known to inhibit osteoclastic formation by a cell-to-cell contact via cytotoxic T lymphocyte antigen 4 (CTLA4) [54].

Bisphosphonates are able to modify immune cell activities. This was particularly demonstrated with *γδ*-T cells. These cells represent 5% of CD3^+^ T cells in human peripheral blood and most of them belong to the V*γ*9V*δ*2 subset. Their name is based on the fact that they express a heterodimeric T cell receptor (TCR) composed of *γ* and *δ* chains, in contrast with the classic TCR, composed of *α* and *β* chains [56]. These cells were detected in rheumatoid arthritis patients and were shown to be capable of secreting IL-17 and IFN-*γ* according to environmental cues [57]. It has also been demonstrated that N-BPs such as Zoledronate could induce IFN-*γ* production by *γδ* T cells* in vitro* and* in vivo* [19]. This activation is likely to be due to the inhibition of farnesyl-diphosphate synthase by N-BPs that would lead to the accumulation of isopentenyl diphosphate and dimethylallyl diphosphate, which are two agonists of V*γ*9V*δ*2-TCR [19]. Therefore, *γδ* T cell stimulation may potentiate the antiresorptive effects of N-BPs.

In a recent study, Komatsu et al. (2014) [58] have unraveled the link between Th17 cells and Treg cells. They showed that, in arthritic conditions, CD25^+^CD4^+^Foxp3^+^ T cells lost FoxP3 expression and went through a “transdifferentiation” process into Th17 cells, induced by the synovial fibroblasts-derived IL-6. These ex-FoxP3 Th17 cells had more pronounced osteoclastic effects than naïve CD4^+^T cell-derived Th17 cells. They were also characterized by the expression of Sox4, CCR6, CCL20, IL-23 receptor (IL-23R), and RANKL [58].

A new therapy for rheumatoid arthritis based on CTLA-4 immunoglobulin underlined also the connection between bone and immune system. On the one hand, CTLA-4 immunoglobulin was used to suppress immune responses by targeting T lymphocyte activation antigens CD80/86 on antigen-presenting cells and thus blocking the costimulation [59]. On the other hand, Bozec and collaborators have shown that CTLA-4 immunoglobulin induced the activation of indoleamine 2,3-dioxygenase (IDO) in osteoclast precursors. IDO is known to metabolize tryptophan, promote apoptosis, and, therefore, decrease bone destruction [60].

Bone remodeling is also modulated in inflammation and during early responses of immune system. Toll-like receptors 2 (TLR2) and 4 (TLR4) activation by pathogen-associated molecular patterns (PAMPs) or damage-associated molecular patterns (DAMPs) in macrophages could stimulate TNF-*α* production and, thus, bone resorption [61].

Cathepsin K is a cysteine protease that is highly expressed by osteoclasts. It degrades type I collagen, which is required to adsorb calcium hydroxyapatite and leads to bone resorption [62]. The cathepsin K-specific inhibitor NC-2300 was initially developed to suppress bone destruction, but it has also shown anti-inflammatory properties when tested in animal model of rheumatoid arthritis. Indeed, cathepsin K participates in TLR9 activation in dendritic cells and stimulated IL-6 and IL-23 production [63]. A recent study used NC-2300 to treat periapical lesion in a rat model and they observed a reduction of inflammation and bone destruction [64]. Odanacatib, another cathepsin K inhibitor, has been developed for the treatment of postmenopausal osteoporosis with encouraging results [65].

## 4. Recent Advances in MRONJ Treatments

Treatments of MRONJ depend on multiple variables such as age, sex, disease status, ONJ stage, comorbidities, and symptoms. Globally, two approaches are currently considered in clinical practice: (1) conservative nonsurgical and (2) surgical procedures. Conservative management focuses on maintaining optimal oral hygiene (home self-care and professional dental care), elimination of active dental and periodontal disease, topical antimicrobial mouth rinses (chlorhexidine 0.12%), and systemic antibiotic therapy. When required, surgical therapy consists of a surgical debridement and/or resection covered by a full-thickness mucoperiosteal flap. Indeed, surgery provides pain control and infection control, relieves soft tissue irritations, and decreases osteolysis [7, 66]. Beside these approaches, several treatments have been developed and used to treat MRONJ:

- *Teriparatide*: as we previously mentioned, Teriparatide is a recombinant human parathyroid hormone, which has stimulatory effects on osteoblasts and osteoclasts and leads to an increase in bone turnover and bone formation, as an osteoanabolic agent [67]. This treatment has shown encouraging results in MRONJ patients [30] and might be recommended in future years in osteoporotic patients without cancer or radiation therapy, for a short-time therapy. Indeed, preclinical studies demonstrated an increased risk of osteosarcoma with long-term therapy [67].
- *Bone morphogenetic proteins (BMPs)*: another potential treatment concerns the use of bone morphogenetic proteins (BMPs), a subgroup of the transforming growth factor-*β* (TGF-*β*) family. BMPs are implied in bone and cartilage formation during development and growth [68]. Among them, BMP-2 and BMP-7 are recognized as effective bone formation inducers and have been approved by the Food and Drug Administration (FDA) in 2001-2002 for this therapeutic indication [69]. They have been used in orthopedics and oral/maxillofacial surgery, including MRONJ [43]. In this clinical situation, BMP-2 was delivered during surgery in the cleaned bone cavity, inducing a successful healing of the necrotic area and new bone formation. Unfortunately, BMPs could exhibit important side effects such as inflammation, bone resorption, swelling, seroma, and carcinogenic effects but these side effects could be dose-dependent [69]. Nevertheless, future well-designed randomized clinical trials are needed to ascertain the safety and efficacy of BMPs.
- *Platelet concentrates*: the use of autologous platelet concentrate as a topical agent during bone resection could also constitute a promising therapeutic strategy. These concentrates are composed of human platelets and are thus enriched in growth factors such as platelet-derived growth factor (PDGF), transforming growth factor-*β* (TGF-*β*), vascular endothelial growth factor (VEGF), and epidermal grow factor (EGF) [70]. Dohan Ehrenfest et al. [71] defined four groups of platelet concentrates based on their fibrin and leucocyte content: leucocyte and platelet-rich fibrin (L-PRF); leucocyte and platelet-rich plasma (L-PRP); pure platelet-rich plasma (PRP); and pure platelet-rich fibrin (P-PRF). PRP is the most used agent in the prevention and treatment of MRONJ [72]. Nevertheless, L-PRF showed also promising benefits [73] as leucocytes present in L-PRF can address infection and regulate the immune system [74] while physiological fibrin matrix is easier to manipulate during surgery and concentrate the growth factors [75]. Autologous platelet concentrates appear more and more to constitute a valuable implementation for surgical procedures, although no specific guidelines are available so far. The setup of those guidelines is still restrained by the lack of standardized parameters and biological properties of these platelets concentrates, according to various preparation techniques [76]. Another problem is to keep theses factors at grafted site and, thus, the treatment might be variable between patients.
- *Platelet-derived growth factor-BB (PDGF-BB)*: platelet-derived growth factor-BB (PDGF-BB) is a new key factor involved in angiogenesis and osteoformation. It is secreted by preosteoclasts and promotes CD31+ and Emcn+ (Endomucin) vessel subtype formation, as well as MSC and endothelial progenitor cell migration [77]. Treatment with PDGF-BB has been shown to increase vasculogenesis and bone formation in ovariectomy-induced osteoporotic mouse model [78]. This new molecule may constitute a new target in bone resorptive pathology.
- *Low-level laser therapy (LLLT)* with Nd: YAG laser or GaAlAs diode laser has been reported to be useful in the treatment and prevention of MRONJ in association with conservative and surgical management [79–81]. LLLT mechanism of action seems to be photochemical: the photon energy absorbed is converted into metabolic energy that will be used to produce proteins and mitoses [82, 83]. LLLT provides an improvement in vascularisation of mucous membrane, bone regeneration, and pain reduction. It may constitute a safe and effective adjunct therapy but it is not recommended yet as a monotherapy.
- *Hyperbaric oxygen (HBO)* is an effective technique mainly used in difficult healing situation. This healing effect is attributed to the increasing oxygen concentration, immunologic regulation, and reactive oxygen species (ROS) and reactive nitrogen species (RNS) production [84–86]. HBO gives a fast wound healing and pain and swelling reduction in the treatment of MORNJ [86–88]. A randomized controlled trial of HBO therapy in MRONJ, by Freiberger et al. 2012, came to the conclusion that HBO seems to be a useful adjunct to ONJ treatment, especially in severe cases [89].
- *Medical ozone therapy (MOT)* has been demonstrated as antimicrobial, wound healing, vasculogenic, and immunostimulating therapy [90, 91]. MOT acts by preserving the endogenous antioxidant system and by blocking xanthine/xanthine oxidase system [91]. It has been used as an adjuvant treatment in MRONJ cases with a reduction of 90% of the symptoms and the authors indicate that MOT is not a substitute of recommended treatment [92, 93].

For the moment, all these new therapies are adjunctive therapies. Likewise, due to the lack of data or the limited number of studies, it is not possible to evaluate the real effectiveness of those treatments. MRONJ remains a complex and a noneffectively treatable disease. This situation could be a direct consequence of the lack of molecular understanding that is mandatory in order to elaborate an efficient pharmacological agent. This could be the reason why MSCs were investigated in multiple studies with a dual objective: (1) cellular therapy and (2) a tool to identify new molecular targets favoring the bone reconstruction in such a disease.

## 5. Mesenchymal Stem Cell Therapy in MRONJ

MSCs are multipotent stem cells that are increasingly used in regenerative medicine. MSCs contribute actively to organogenesis during embryogenesis and, thereafter, to the maintenance of adult tissues. To be considered as MSCs, cells must present three characteristics: (1) adherence to plastic culture dishes; (2) expression of CD105, CD73, and CD90 but absence of expression of CD34, CD45, CD14 or CD11b, CD79a or CD19, and HLA-DR markers; and (3) capacity to differentiate into osteoblasts, chondrocytes, and adipocytes [94]. In adulthood, MSCs are usually isolated from bone marrow or adipose tissue but are also present in various other tissues [95]. Due to their ability to differentiate into osteocytes but most importantly due to the recent characterization of their immunomodulatory properties, MSCs should strongly be regarded as grafting material in osteonecrosis foci [96].

### 5.1. Immunomodulatory Effects of MSCs

Three different cells together constitute the innate immune system: natural killer (NK) cells, dendritic cells (DCs), and macrophages. NK cells are involved in antiviral or antitumoral defense and are known to kill infected or tumor cells without MHC1 restriction [97]. MSCs are able to inhibit NK cells proliferation by secreting indolamine 2,3-dioxygenase (IDO) and prostaglandin E2 (PGE2) [98]. DC are antigen-presenting cells that link the innate and adaptive immune system [99]. MSCs exert an inhibitory effect on dendritic cells (DCs) differentiation through soluble factors (IL-6, PGE2) and cell-to-cell contact [100, 101]. Macrophages are monocyte-derived phagocytic cells that are basically divided into two categories: M1 macrophages promote proinflammatory reaction, and M2 macrophages are rather involved in wound healing [102]. There is a temporal relation between M1 and M2 macrophages: inflammatory M1 macrophages are predominant at early stages of tissue lesion and, later, wound healing M2 macrophages become predominant. M1 macrophage stage is necessary to clear dead tissue or infectious agents, and M2 macrophage stage allows the resolution of the injury [103]. This balance between these two phenotypes constitutes an important target in future therapies and can be influenced by MSCs. Interestingly, MSCs support M2 macrophages proliferation over M1 macrophages by increasing IL-4 and IL-13 levels and by decreasing TNF-*α* and IL-6 levels [104].

T cells and B cells are the actors of the adaptive immune system. Although the effect of MSCs on B cells is still unclear, some studies support that MSCs are able to inhibit B cell differentiation and to decrease immunoglobulin production [105]. However, those results are still questioned [106]. The role of MSCs in modulating T cells depends on T cell subtypes. MSCs are indeed able to inhibit cytotoxic T lymphocyte proliferation and generate regulatory CD8^+^ cells in coculture conditions [107]. It has also been demonstrated that MSCs support regulatory T (Treg) cell proliferation by secreting Il-10, PGE2, and TGF-*β* and by promoting the expression of Foxp3 [108]. Globally, MSCs inhibit Th1, Th2, and Th17 proliferation. However, it has also been demonstrated that grafting MSC could increase Th2 activity in non-Th2-dominated autoimmunity and in allotransplant models [109]. The exact mechanism by which MSCs suppress T helper cell proliferation is poorly understood, but interesting theories are rising up. Indeed, Sato et al. suggested that MSCs secrete nitric oxide (NO), which would suppress STAT5 phosphorylation and T helper cell proliferation [110]. Meisel et al. showed that IDO, which catalyzes the conversion from tryptophan to kynurenine, was produced by MSCs and could be a T cell inhibitory effector pathway [111]. Finally, it is important to underline that, in those conditions, MSCs needed to be previously activated by IFN-*γ* to exert their immunomodulatory effects which could also be linked to their effects on inflammation [106, 112].

### 5.2. Therapeutic Effects of MSC in MRONJ

Several studies exploited these interesting MSC-related immunomodulatory effects in the treatment of MRONJ. MSC grafts have been performed in mice, pigs, and humans with encouraging results [113–115]. Of note, the key of the success obtained with MSCs seems to be more linked to their immunomodulatory properties rather than their possible osteoblast differentiation. Indeed, the efficacy of MSC grafts is dependent on their capacity to decrease levels of IL-17, IL-6, c-reactive protein, and Th17 cells and to increase levels of IL-10, TGF-*β*1, and Treg cells [113, 114]. So, although MSCs are able to differentiate into osteoblast precursors [100], there is no evidence that grafted MSCs directly participate in bone regeneration by osteoblast differentiation. Indeed, median survival of grafted MSCs is about one or two weeks: this timeframe is not consistent with a direct bone regeneration but is more related to an indirect effect [116]. Moreover, the absence of direct participation of grafted MSCs in tissue regeneration by differentiation has also been reported in various neurological diseases models where grafted MSCs did not last at the transplantation site, despite their beneficial effects on neuronal regeneration [117]. All these observations led to the hypothesis that MSCs beneficial effects are due to their capacity to secrete cytokines and/or growth factors, gathered under the term “*secretome.*” Furthermore, from this point of view, this MSC secretome should promote endogenous tissue regeneration, apoptosis inhibition, and angiogenesis. Therefore, MSC-conditioned culture medium has been injected into rat MRONJ-like model, and full recovery was indeed observed in 63% cases. Histological analyses showed that MSC secretome exhibits anti-inflammatory, osteogenic, angiogenic, and antiapoptotic properties [118].

On the other hand, it has been demonstrated that the jaw bone cells arise from cranial neural-crest stem cells (NCSCs) [35]. Furthermore, using cell tracing or mapping strategies [36], it has been recently demonstrated that jaw bone defects are healed through neural-crest cell recruitment. Based on these observations, grafting NCSCs or NCSC secretome in MRONJ cases might be a new and relevant therapeutic strategy to investigate. Interestingly, it was recently demonstrated that adult bone marrow MSCs, but also adult adipose tissue derived MSCs, are heterogeneous populations containing NCSCs as well. The use of pure populations of MSCs or NCSCs (or their respective secretomes) therefore deserves to be considered in MRONJ animal models.

Altogether, although grafted MSCs/NCSCs are not directly responsible for bone regeneration observed in MRONJ animal models, they are suspected to recruit and stimulate local or regional endogenous cells to differentiate into osteoblasts and thus bone formation (1). Knowing the beneficial effect of grafted MSC in MRONJ, we can suggest that MSC would have an impact on bone remodeling (2) which is targeted by BPs and denosumab. As described before, MSCs exhibit immunomodulatory properties (3) and seem to decrease the inflammation in MRONJ. In this context, it would therefore be useful to recapitulate the molecular regulatory events controlling osteogenic differentiation, bone remodeling, and osteoimmunology.

## 6. Conclusions

Despite the recent advances in MRONJ pharmacological treatments, we are still looking forward to a curative treatment without dangerous side effects and an understanding of MRONJ physiopathogenesis. During last decade, cell therapy was considered to be efficient for treating different medical conditions and, in this view, MSC grafting in MRONJ is a recent therapeutic strategy that shows good results. However, after first clinical trials, cell therapy of MRONJ using MSC has to get back to the laboratory in order to understand their effects, at the molecular and the cellular levels as little information is available concerning the MSC mechanisms of action. Research studies should therefore now focus on the molecular mechanisms underlying the beneficial effects of MSC grafts. Likewise, it is unclear if the major impact of this cellular therapy is immunological or bone related. This unclear situation is possibly related to the fact that MRONJ is not a simple bone disease but could represent a model of osteoimmunology pathology similar to what we observed in rheumatoid arthritis. This could explain the absence of a strictly defined treatment and a proper characterization of MRONJ physiopathology. We strongly believe that a molecular dissection of grafted MSC effects in MRONJ would allow us to get a better understanding of the physiopathological sequences in this clinical situation but also to design new pharmacological approaches to help patients.

## Figures and Tables

**Table 1:** Drugs demonstrated to be implied in the triggering of the jaw osteonecrosis: name, mode of action or molecular target, and therapeutic indications.

Drugs	Mode of action	Indications
Biphosphonate: SBPs N-BPs	Nonhydrolysable cytotoxic analogs of ATP Farnesyl-diphosphate (FPP) synthase inhibition	Osteoporosis Paget's disease Hypercalcemia of malignancy Tumor-associated osteolysis

Denosumab	Monoclonal antibody that inactivates RANKL	Osteoporosis Tumor-associated osteolysis

Bevacizumab	Monoclonal antibody that inactivates VEGF	Glioblastoma Metastatic cancers: breast, renal, lung, colorectal

Sunitinib, Sorafenib, Cabozantinib	Tyrosine kinase inhibitors that block VEGF receptor	Metastatic cancers: breast, renal, lung, colorectal

Everolimus, Temsirolimus®	mTor inhibitors	Metastatic renal cell carcinoma
